# North Carolina Toxic Substance Incidents Program 2010–2015: Identifying Areas for Injury Prevention Efforts

**DOI:** 10.3390/toxics5030016

**Published:** 2017-08-06

**Authors:** Samantha Eiffert, Suze Etienne, Annie Hirsch, Ricky Langley

**Affiliations:** North Carolina Occupational and Environmental Epidemiology Branch (NC OEEB), 5505 Six Forks Rd., Raleigh, NC 27699, USA; sam.eiffert@socket.net (S.E.); smetienne19@gmail.com (S.E.); ademuth1@gmail.com (A.H.)

**Keywords:** chemicals, spills, transportation, industry, injuries, evacuation, explosion, carbon monoxide

## Abstract

The National Toxic Substance Incidents Program (NTSIP) is a surveillance system designed to capture acute toxic substance releases, factors contributing to the release, and any associated injuries. North Carolina has participated since 2010, when NTSIP was established. This article will present a descriptive statistical summary from 2010 to 2015 focused on releases that resulted in injuries in order to identify areas for public health prevention efforts. Of the 1690 toxic releases in North Carolina, 155 incidents resulted in injuries and 500 people were injured. Carbon monoxide injured the greatest number of people. Of the incidents that resulted in injuries, 68 occurred at private vehicles or residences (44%), injuring 124 people (25%). Over half of events where at least one responder was injured occurred at private vehicles or residences. Events occurring at private residences did not have a significant relationship between evacuations and injuries, while for industry-related events, the odds of an evacuation being ordered were 8.18 times greater (OR = 8.18, 95% CI = 5.19, 12.89) when there were injuries associated with an event. Intervention efforts should focus on preventing responder injuries while responding to private residence releases and educating the general public on how to prevent injuries by self-evacuating areas where hazardous chemicals have been released.

## 1. Introduction

The purpose of the National Toxic Substance Incidents Program (NTSIP) surveillance system is to identify areas and factors leading to injuries and/or evacuations so these types of events can be prevented in the future. NTSIP replaced a previous surveillance system called the Hazardous Substances Emergency Events Surveillance (HSEES) Program [1]. NTSIP was designed to expand the work done under the HSEES program and provide a more comprehensive approach to incident surveillance, prevention, and response [1]. North Carolina, USA, has participated in NTSIP since its establishment in 2010, and previously participated in the HSEES Program. One article that summarized HSEES data from nine states from 1999 to 2008 found that the five chemicals associated with the most injuries were carbon monoxide, ammonia, chlorine, hydrochloric acid, and sulfuric acid [2]. This article also found that the most common locations of events that involved responder injuries were private households followed by merchant wholesalers and utilities. Seventeen percent of all responder injuries were due to releases involving illegal methamphetamine laboratories, and the odds of a shelter-in-place or an evacuation order being given were higher in incidents with injuries compared to events without injuries [2]. An article published using HSEES data from the state of New York from 1993 to 2002 found that the six most common chemicals associated with injuries were ammonia, pyridine, carbon monoxide, hydrochloric acid, sodium hypochlorite, and sulfuric acid [3]. Several articles have been written summarizing HSEES data across states and for individual states [1,2,3,4,5,6,7,8,9], but there are no articles currently published that examine NTSIP-eligible releases in detail from any one state. While there are NTSIP publications focused on one chemical or one release event, there are no publications summarizing the entirety of NTSIP data. For an example, a recent NTSIP publication focuses on first responder and general public injuries in the case of chemical suicides for NTSIP participating states [10]. There may be more value in examining data for individual states as some states may have more releases of a certain chemical that the aggregate data for all participating states might not show. This knowledge can help direct targeted public health interventions that result in a greater impact at the state level. North Carolina has used state level NTSIP data to justify several prevention programs. Funding for the NTSIP program will end in September of 2017, and results from this study may help identify new types of chemical releases or exposures not found in older studies. In this paper, we provide an overview of the acute toxic substance releases in North Carolina from 2010 to 2015, with a focus on events that led to one or more injury, to identify areas for public health interventions.

## 2. Methods

North Carolina collects toxic substance release data according to NTSIP guidelines. NTSIP captures acute toxic substance releases, defined as emergency, illegal or uncontrolled releases of a toxic substance lasting 72 hours or less. NTSIP has a defined list of “any quantity” toxic substances to be reported in the database. (The any quantity reporting list includes 724 chemicals that appear at least twice in the following sources: Emergency Response Planning Guidelines, American Industrial Hygiene Association, Risk Management Plan Chemicals, EPA Office of Emergency Management, 40 CFR 68, two ATSDR master lists of chemicals that could be used as weapons of chemical terrorism, Scientific Working Group on Forensic Analysis of Chemical Terrorism Draft List of Prioritized Chemicals, FBI, and the Department of Homeland Security Chemical Facilities Anti-Terrorism Standard Appendix A chemicals [11].) Mercury, sodium hydroxide, and carbon monoxide (above 50 ppm) are also included in the any quantity reporting list based on the number of victims in HSEES [11]. NTSIP also has a list of 105 substances to be reported if the quantity released is greater or equal to one pound, based on an EPA reporting requirement [12]. Some less toxic substances are included in the NTSIP database at higher quantities; for example, paint is included if the release is 100 gallons or greater [11]. 

If a substance is considered toxic (A toxic substance includes “any element, substance, compound, or mixture including disease-causing agents, which after release into the environment and upon exposure, ingestion, inhalation, or assimilation into any organism either directly from the environment or indirectly by ingestion through the food chain, will or may reasonably be anticipated to cause death, disease, behavioral abnormalities, cancer, genetic mutation, physiological malformations including malformations in reproduction or physical deformation in such organisms or their offspring” [11].) but is not on either of the two NTSIP reporting lists, it is reported if the release is greater than or equal to ten pounds or one gallon. Petroleum products are only reported if there is an injury or public health action. A public health action includes evacuation, health advisory, well survey, alternate water, fishing ban, prohibition of consumption of produce or livestock, health investigation, shutdown of water intakes, and environmental sampling [11]. The NTSIP database focuses on capturing unintentional acute chemical releases that occur outside of private citizen properties. However, if a toxic chemical release occurs in a private residence or vehicle and results in a public health action, this is recorded in the NTSIP database. Toxic substance release incidents are identified through multiple data sources including the U.S. Department of Transportation, Carolinas Poison Center, National Response Center, North Carolina Emergency Management, and media reports. 

For this descriptive study, data from North Carolina’s NTSIP surveillance system was used to provide a descriptive overview of toxic release incidents and corresponding injuries. Data from 2010 to 2015 was used. This range of dates was selected because the NTSIP program began in 2010, and at the time of data analysis, all years through 2015 had been entered into the database and the accuracy of the entered events had been verified through a quality control process. Descriptive statistics were performed on key variables. Variables that were related to cause of release or injury were included in the analysis, while some ancillary variables were excluded, as they were either not relevant to injuries or were too detailed and would have encumbered the focus of the paper. After performing exploratory data analysis, frequency analyses, percentages and odds ratios were performed using SAS 9.4 (Cary, NC, USA). Taylor series 95% confidence intervals were used for interpreting the odds ratios. 

## 3. Results

### 3.1. Overview

From 2010 to 2015, 1690 NTSIP-eligible toxic release incidents were captured in North Carolina’s database (Table 1). Most incidents involved only one chemical, but 38 incidents involved two or more chemicals. Most releases were spills (*n* = 1222, 71%), followed by volatilization, aerosolization, or vapor releases (*n* = 455, 26%), fires (*n* = 24, 1.4%), explosions (*n* = 17, 1%), and radiation (*n* = 2, 0.1%). Natural gas was the most common chemical released (*n* = 138, 8%), followed by sodium hydroxide (*n* = 114, 7%), methamphetamine production related chemicals (*n* = 110, 6%), and ammonia (*n* = 86, 5%).

### 3.2. Victims 

There were 500 victims injured due to acute toxic chemical releases in North Carolina (Table 2). Of the 1690 total events, 155 events (9%) resulted in one or more injuries, with one event resulting in 21 injuries. Of the 397 victims for whom sex was known, 240 (60%) were male and 157 (40%) were female. Of the 470 victims for whom age was known, most were adults age 18 or older (*n* = 418, 89%), while 52 (11%) were children ages 0–17. A total of 836 injuries were recorded among the 500 victims. Most victims (*n* = 289, 58%) sustained one injury, however some victims had up to six injuries associated with the chemical release. The most common injuries were respiratory system problems (*n* = 228, 46%), followed by headaches (*n* = 149, 30%), gastrointestinal issues (*n* = 134, 27%), and central nervous system symptoms (*n* = 133, 27%) (Table 2). That respiratory system problems and headache were the most common injuries is not surprising as the top five chemicals released are known respiratory irritants or known to cause headaches. Employees were the most commonly injured (*n* = 253, 51%), followed by the general public (*n* = 169, 34%) and police officers (*n* = 36, 7%). Most victims (*n* = 279, 56%) were treated at a hospital but not admitted. Seventy-one victims (14%) were treated on the scene with first aid and 57 (11%) were treated at the hospital and admitted. Evacuations were ordered for 299 (18%) of events, 75 of which had associated injuries. 

During the study period, 20 people (4%) died either on the scene or upon arrival at a hospital (Table 3). There were ten unintentional deaths due to chemical releases; three of these deaths were industry-related and seven occurred in private residences. The industry-related deaths were due to carbon monoxide poisoning, a propane explosion, and a natural gas explosion. Of the seven unintentional deaths that occurred at private residences, three were drug related deaths and four were carbon monoxide poisonings. 

### 3.3. First Responder Injuries

The most frequent responders to chemical releases were company response teams (*n* = 724, 43%), followed by fire departments (*n* = 389, 23%) and law enforcement agencies (*n* = 327, 19%). A total of 69 first responders were injured in 31 chemical releases (Table 4). Responders sustained a total of 105 injuries; the most common responder injuries were respiratory system problems (*n* = 29, 28%), central nervous system problems (*n* = 18, 17%), and gastrointestinal issues (*n* = 15, 14%). Police officers were injured most often (*n* = 36, 52%), followed by firefighters (*n* = 17, 25%), unspecified responders (*n* = 7, 10%), EMS personnel (*n* = 5, 7%), company response team personnel (*n* = 3, 4%), and third party clean-up contractors (*n* = 1, 1%). There were nine methamphetamine production related chemical releases that injured 20 (29%) responders, followed by three carbon monoxide releases which injured 17 (25%) responders and one sulfuric acid release which injured 6 (9%) responders. Out of the 20 first responders injured due to methamphetamine production related chemicals, 17 (85%) were law enforcement officers. Sixteen (52%) of the 31 events where at least one responder was injured occurred at private vehicles or residences and resulted in 32 (46%) injured first responders, 25 of which were law enforcement officers. Fourteen law enforcement injuries that occurred at private vehicles or residences were methamphetamine production related chemical injuries. All but one law enforcement officer injury due to methamphetamine production related chemicals occurred at a private vehicle or residence; the other event occurred at a motel. The odds of first responder injuries when a chemical release occurred at a private residence or vehicle was nine times the odds of first responder injuries at an industry location (OR = 8.99, 95% CI = 4.37, 18.49). 

### 3.4. Primary Causes of Toxic Releases

Most releases were attributed to human error (*n* = 1008, 60%), followed by equipment failure (*n* = 436, 26%). Of events that resulted in injuries, human error was the primary factor in 65 events (42%) and resulted in 255 injured people (51%). Illegal acts was the primary factor in 36 events (23%) that resulted in one or more injury and 73 (15%) of victims were injured due to an illegal act. Equipment failure was the primary factor in 29 events (19%) that resulted in one or more injury and injured 115 (23%) people. Illegal acts was the primary factor in 23% of events but accounted for 15% of the total number of people injured; illegal acts were more likely to occur in private vehicles or residences (of the 36 illegal acts that resulted in injuries, 31 occurred in private vehicles or residences). The odds of an event resulting in an injury due to human error (OR = 0.45, 95% CI = 0.32, 0.63) or equipment failure (OR = 0.64, 95% CI = 0.42, 0.97) were less than the odds of an event resulting in one or more injury when the release was due to illegal activity (*n* = 124) (OR= 4.97, 95% CI= 3.23, 7.65). (Note: the reference group for each of these odds ratios is the odds of injury due to all other causes for each respective odds ratio (that is, the odds of an event resulting in an injury due to human error is 0.45 times the odds of an event resulting in an injury not due to human error, and so on.)) 

### 3.5. Events Occurring at a Private Vehicle or Residence

While the NTSIP database primarily captures toxic substance releases that do not occur in a private vehicle or residence, 192 (11%) events were captured that occurred at a private vehicle or residence. Of these events, 104 events (54%) were associated with an illegal act and 52 events (27%) were due to human error. Illegal acts was the primary factor in over half of releases that occurred at private vehicles or residences. Eleven releases (6%) were intentional acts; 10 of these were chemical suicides. Out of the 155 chemical releases that resulted in one or more injury, 68 events occurred at a private vehicle or residence (44%) and 124 people (25%) were injured. Just over 10% of chemical release events occurred at a private vehicle or residence, but these events contributed to one quarter of injuries. Methamphetamine chemicals injured 49 (40%) people at private vehicles or residences, carbon monoxide injured 28 (23%) people, and natural gas injured 3 (2%) people. The odds of a release at a private vehicle or residence resulting in one or more injury was 8.89 times the odds (OR = 8.89, 95% CI = 6.16, 12.83) of a release that was not at a private vehicle or residence resulting in one or more injury. When the odds of general public injuries and first responder injuries are compared for events occurring at a private vehicle or residence, both the general public (OR = 26.12, 95% CI = 15.29, 44.63) and first responders (OR = 8.99, 95% CI = 4.37, 18.49) have higher odds of injury when an event occurred at a private vehicle or residence than when the event was industry-related. However, there were more people injured during industry-related releases. One third of events occurring at a private vehicle or residence resulted in an official evacuation (*n* = 67, 33%).

### 3.6. Evacuations

Seventy-three events injured members of the general public. Among the 155 incidents with injuries, 75 led to an official evacuation. The odds of an evacuation being ordered when there were one or more injuries was 5.62 (OR = 5.62, 95% CI = 3.97, 7.95) times the odds of an evacuation being ordered when there were no injuries. However, when this is stratified by whether the event occurred at a private vehicle or residence, events occurring at private residences did not have a significant relationship between evacuations and injuries, while for events that were industry-related, the odds of an evacuation being ordered were 8.18 times greater (OR = 8.18, 95% CI = 5.19, 12.89) when there were one or more injuries associated with an event than when there were no injuries associated with an event. Evacuations were more likely to occur when the release was industry-related. The odds of sheltering in place was 2.89 times greater when there was one or more injury than when there were no injuries (OR = 2.89, 95% CI = 1.35, 6.17).

### 3.7. Fixed Facilities or Transportation

Events were just as likely to occur at a fixed facility (*n* = 841, 50%) or during transportation (*n* = 849, 50%). However, 125 fixed facility events resulted in one or more injury, while 30 transportation events resulted in injuries. For events that did not occur at private vehicles or residences, the odds of a fixed facility event resulting in one or more injury was 5.42 times the odds of a transportation event resulting in one or more injury (OR = 5.42, 95% CI = 3.16, 9.31). 

### 3.8. Chemicals Resulting in Injuries

Top chemicals involved in injuries were also analyzed, including natural gas, sodium hydroxide, methamphetamine chemicals, sulfuric acid, carbon monoxide, and hydrochloric acid (Table 5). Carbon monoxide led to the greatest number of unintentional deaths (*n* = 5) and the greatest number of injured people (*n* = 137, 27%) despite having relatively few releases (*n* = 40, 2%). Natural gas, the most common chemical released (*n* = 138, 8%), injured far fewer people (*n* = 28, 6%) and claimed one life. The odds of injury due to a carbon monoxide release were greater than all other chemicals (OR = 27.98, 95% CI = 13.89, 56.35), and the odds of injury due to hydrochloric acid was 3.58 (OR = 3.58, 95% CI = 1.71, 7.45) greater than other chemicals. The odds of injury due to methamphetamine chemicals for events that did not occur at a private vehicle or residence were 10.04 (OR = 10.04, 95% CI = 2.36, 42.73) times the odds of injury due to other chemicals. However, there were only eight total events involving methamphetamine chemicals that did not occur at a private vehicle or residence. 

## 4. Discussion

The primary purpose of North Carolina’s acute toxic chemical release surveillance system is to identify which populations are most at risk for injuries due to these releases and areas where public health prevention activities can be implemented to prevent injuries and improve health. The odds of injury due to a carbon monoxide release were significantly greater than all other injuries due to chemical releases. However, it is likely that the data collection methods artificially inflated the odds of injury. NTSIP only captures carbon monoxide releases if the concentration is known to be greater than or equal to 50 ppm, and carbon monoxide releases that occur in a private vehicle or residence are only included if they led to a public health action. Carbon monoxide events involving faulty heaters in homes or suicides where only one person is exposed are not included in the database. Also, since carbon monoxide is odorless and colorless, these releases are often not detected until injuries occur. The same artificial inflation is most likely occurring for natural gas as well. Natural gas and other petroleum product releases are only included in the NTSIP database if there is a public health action. This could explain the very high odds of an evacuation being ordered when the chemical release was natural gas compared to other chemicals. However, both carbon monoxide and natural gas releases have been shown to be dangerous releases that can result in injuries. The main limitation of this study is that data reported is specific to the state of North Carolina. While the findings may be generalizable to other similar states, the results of this analysis may not apply to states where climate or industry is vastly different from that of North Carolina. For an example, some of the unintentional carbon monoxide poisonings were due to people bringing grills indoors to stay warm. States with warmer climates may not have this challenge, and states with more industry may see more injuries due to industry-related chemical releases. Another limitation is that the number of toxic release events that resulted in regulatory actions such as citations, fines, or license suspension is unknown. 

Carbon monoxide injured the greatest number of people, followed by methamphetamine production-related chemicals, sulfuric acid, natural gas, and isopropanol. Both carbon monoxide and sulfuric acid were listed as two of the top five most common chemicals associated with injuries in the literature [3,4]. In order to assist North Carolina state partners in identifying populations at risk of carbon monoxide poisoning and guide intervention and prevention strategies, the Occupational and Environmental Epidemiology Branch (OEEB) began issuing a monthly carbon monoxide poisoning surveillance report in December 2013 using data from the Carolinas Poison Center and emergency department visit data from NC DETECT, North Carolina’s statewide syndromic surveillance system. These monthly reports are sent to local health directors, emergency management coordinators, and other stakeholders. In December 2016, work-related carbon monoxide poisoning became a reportable condition in North Carolina. 

The odds of injury due to an illegal act were significantly higher than the odds of injury due to other primary factors. Illegal acts are more likely to occur at private vehicles or residences, which may explain why half of events that led to first responder injuries occurred at private vehicles or residences, nine of which were methamphetamine production related. First responders had higher odds of injury when responding to releases that occurred at private vehicles or residences than when responding to industry-related events. When responding to a private vehicle or residence, there may not be much, if any, information available on potentially hazardous chemicals present in the residence or vehicle, especially if illegal activities are occurring. In order to address first responder injuries at private vehicles and residences, North Carolina has started an awareness training program for public workers that may visit homes where opiates and/or methamphetamine is being produced and what precautions to take.

While just over 10% of NTSIP eligible releases occurred at a private vehicle or residence, these releases were responsible for one quarter of all injuries, suggesting that events resulting in injuries are more common when releases occur in a private vehicle or residence. There was no significant relationship between evacuations and injuries for events that occurred at a private vehicle or residence. People may be less likely to evacuate an area if an event occurs at a private vehicle or residence even if a substance poses a human health risk due to the nature of criminal activity, or situations may have escalated past people’s ability to evacuate (such as carbon monoxide poisonings rendering victims unconscious). There was a significant difference in the number of victims injured at fixed facilities, which is not surprising as there are often more people in close proximity to releases at fixed facilities than in transportation, which may only affect one or two people. 

North Carolina used chemical release data from both HSEES and NTSIP to develop a Chemical Release Investigation Kit and Template (CRIKT) for the most commonly released chemicals. Kits were created for 35 frequently released chemicals to ensure that local health departments have access to accurate chemical information and the best response practices to protect public health in the event of a chemical release. Each CRIKT is available online and includes a fact sheet, a response guide, and a line listing template. 

## 5. Conclusions

To reduce morbidity and mortality due to acute toxic chemical releases in North Carolina, intervention efforts should continue to focus on carbon monoxide poisoning prevention and education. These results confirm other published results from both New York and aggregate results from nine states that carbon monoxide releases are a common source of injury. However, these results show that new efforts should focus on first responder injury prevention when responding to private vehicle or residence releases and general public education on common hazardous substances and how to reduce potential injuries by self-evacuating areas where hazardous chemicals have been released. An area of interest for future research is to assess fixed facilities that utilize toxic chemicals and the population centers near them in order to assess the risk to vulnerable populations such as nursing homes, hospitals, and schools. Funding for the NTISP program is scheduled to end in September of 2017. However, North Carolina plans to continue toxic substance release surveillance as this data can be important for successfully targeting intervention efforts in order to make the greatest impact on injury prevention. 

## Figures and Tables

**Table 1:** NTSIP-eligible toxic substance releases information for North Carolina, 2010–2015.

**NTSIP-Eligible Releases**		**Chemical-Related Fatalities**	
Toxic substance releases total	1690	Unintentional chemical-related fatalities	10 (0.6%)
Releases that led to an official evacuation	299 (18%)	Chemical suicides	10 (0.6%)
Releases resulting in one or more injuries	155 (9%)	Primary notification source *	
**Type of release**		U.S. Department of Transportation	836 (49%)
Fixed-facility releases	841 (50%)	Emergency Government Services	369 (22%)
Transportation releases	849 (50%)	National Response Center	252 (15%)
Industry-related releases	1498 (89%)	Media	152 (9%)
Private vehicle or residence releases	192 (11%)	Other Government Agencies	80 (5%)

* one observation was missing a primary notification source.

**Table 2:** Number, type, and severity of injuries that resulted from NTSIP-eligible toxic substance releases, North Carolina, 2010–2015.

**Injury Type ^a^**		**Severity of Injury ^a^**	
Respiratory system problems	228 (46%)	Death on scene or arrival at hospital	20 (4%)
Headache	149 (30%)	Treated at hospital (admitted)	57 (11%)
Gastrointestinal problems	134 (27%)	Treated at hospital (not admitted)	279 (56%)
Dizziness or other CNS symptoms	133 (27%)	Treated on scene (first aid)	71 (14%)
Burns	50 (10%)	Observed as hospital (no treatment)	49 (10%)
Heart problems	47 (9%)	Other category or unknown	24 (5%)
Eye irritation	36 (7%)	**Number of injuries sustained by each victim ^a^**	
Shortness of breath	22 (4%)	One injury	289 (58%)
Skin irritation	20 (4%)	Two injuries	124 (25%)
Chemical-related trauma	10 (2%)	Three injuries	55 (11%)
Heat Stress	5 (1%)	Four injuries	27 (5%)
Other	2 (0.4%)	Five or Six injuries	5 (1%)
Total injuries: 836			
Total injured persons: 500			

^a^ Non-chemical related injuries were not included; percentages are out of total injured persons (*n* = 500).

**Table 3:** Chemicals involved in victim deaths, North Carolina, 2010–2015.

Chemical	Suicide	Unintentional
Carbon Monoxide	1	5
Hydrogen Sulfide	4	
Mixture: Calcium sulfide (Ca(Sx)), Nitric Acid NOS	1	
Mixture: Gluconic Acid, Hydrochloric Acid, Hydrochloric Acid NOS, Hydrogen Sulfide NOS, Vanadyl Sulfate	1	
Methamphetamine Chemicals NOS		2
Natural Gas		1
Propane		1
Reaction: Calcium sulfide (Ca(Sx)), Drain Cleaner NOS, Hydrogen Sulfide	1	
Reaction: Calcium sulfide (Ca(Sx)), Hydrochloric Acid, Hydrogen Sulfide	1	
Bath Salts (illegal)		1
Reaction: Aluminum Sulfide, Germanium Oxide, Hydrogen Sulfide, Sulfur NOS	1	
Total deaths	10	10

**Table 4:** Responses to chemical releases, North Carolina, 2010–2015

Type of Response	Number of Responses ^a^	Responder Injuries
Company’s response teams	724 (43%)	3 (4%)
Fire department	389 (23%)	17 (25%)
Third party clean-up contractor	251 (15%)	1 (1%)
Law enforcement	327 (19%)	36 (52%)
EMTs	197 (12%)	5 (7%)
Certified hazmat teams	240 (14%)	
Emergency management	224 (13%)	
Dept. of works, utilities, transporation (includs Coast Guard)	95 (6%)	
Other	35 (2%)	7 (10%) ^b^
Environmental agency or EPA	54 (3%)	
Health department or agency	21 (1%)	
Specialized multi-agency team	7 (0.4%)	
Total	2564	69
No Response	265 (16%)	

^a^ Percentages are out of total number of chemical releases (*n* = 1690); ^b^ unspecified responders.

**Table 5:** Chemicals most frequently released and corresponding injuries and evacuations, North Carolina, 2010–2015.

Chemical^1^	Releases	Treated at Hospital (Admitted)	Treated at Hospital (Not Admitted)	Observed at Hospital	Treated on Scene (First Aid)	Other or Unknown	Releases with One or More Victim	Total Victims	Evacuations
Natural gas	138	3	14	0	10	1	10	28	120
Sodium Hydroxide	114	0	3	0	2	0	4	5	4
Methamphetamine Chemicals NOS	110	10	13	1	30	6	32	60	29
Ammonia	86	2	10	2	0	1	5	15	13
Sulfuric Acid	57	0	20	20	0	0	6	40	9
Acetone	45	1	1	0	1	0	2	3	3
Carbon Monoxide	40	11	110	1	8	7	28	137	28
Paint Thinner NOS	40	0	0	0	0	0	0	0	0
Hydrochloric Acid	39	0	11	3	0	4	10	18	9
Hydrogen Peroxide	37	0	0	0	2	0	1	2	2
Potassium Hydroxide	37	0	0	0	0	0	0	0	2
Isopropanol	35	0	21	0	0	0	1	21	1
Mercury	30	0	1	0	0	5	2	6	12
Ink NOS	27	0	0	0	0	0	0	0	0
Chlorine	25	1	12	1	0	3	7	17	10
Methylene Chloride	14	5	4	0	0	0	2	9	1
Propane	17	5	0	0	0	0	3	5	15
Diesel Fuel	15	0	3	0	0	0	3	3	2
Petroleum Distillates	7	0	15	0	0	0	3	15	3
Gasoline	7	1	3	0	3	0	4	7	3
Liquefied Petroleum gas	2	1	1	0	0	0	1	2	2
Petroleum product NOS	1	0	0	0	0	0	0	0	1

^1^ Chemicals that were released as a mixture or reaction are also included as part of this table; NOS = not otherwise specified.
